# From Warm to Cold: Feeding Cold Milk in Preterm Infants with Uncoordinated Oral Feeding Patterns

**DOI:** 10.21203/rs.3.rs-4504972/v1

**Published:** 2024-06-18

**Authors:** Louisa Ferrara, Ranjith Kamity, Zeyar Htun, Vikramaditya Dumpa, Shahidul Islam, Nazeeh Hanna

**Affiliations:** 1 Division of Neonatology, Department of Pediatrics, NYU Langone Hospital - Long Island, NYU Grossman Long Island School of Medicine, Mineola, NY, 11501, USA;; 2 Division of Neonatology, Department of Pediatrics, Arkansas Children’s Hospital, University of Arkansas for Medical Sciences, Little Rock, AR 72205;; 3 Biostatistics Core, Division of Health Services Research, Department of Foundations Medicine, NYU Long Island School of Medicine, Mineola, NY

**Keywords:** Dysphagia, Cold Milk, Oral Feeding, Preterm, Safety, Neonate, Swallow, Coordination

## Abstract

**Objective:**

Premature infants frequently face feeding challenges due to disrupted coordination of sucking, swallowing, and breathing, increasing their risk of dysphagia. There are few effective treatment options available for these infants. In adults experiencing dysphagia, consuming cold foods or liquids can be an effective strategy. This method stimulates the sensory receptors in the pharyngeal mucosa, promoting safer and more effective swallowing. We have previously demonstrated that short-duration feeding (5 swallows) with cold liquid significantly reduces dysphagia in preterm infants; however, the impact of extended cold milk feeding remains unexplored. This study aims to assess the safety of cold milk feedings in preterm infants diagnosed with uncoordinated feeding patterns and its effect on feeding performance.

**Study Design:**

Preterm infants with uncoordinated feeding patterns (n=26) were randomized to be fed milk at either room or cold temperatures using an experimental, randomized crossover design. We monitored axillary and gastric content temperatures, mesenteric blood flow, and feeding performance.

**Result:**

The findings suggest that preterm infants can safely tolerate cold milk without any clinically significant changes in temperature or mesenteric blood flow, and it may enhance certain aspects of feeding performance.

**Conclusion:**

These results suggest that cold milk feeding could be a safe therapeutic option for preterm infants. These results highlight the potential for further comprehensive studies to explore the use of cold milk as an effective therapeutic approach for addressing feeding and swallowing difficulties in preterm infants. Registered at clinicaltrials.org #NCT04421482.

## Introduction

Preterm infants exhibit a developmental trajectory in oral-motor skills from 30 to 45 weeks post-menstrual age (Browne & Ross, 2008). This progression begins with limited coordination of sucking, swallowing, and breathing, eventually leading to a fully integrated sucking pattern. However, during this developmental phase, certain preterm infants may continue to display signs of neurological and physiological immaturity, which can adversely impact their success with oral feeding [1]. An uncoordinated feeding pattern is characterized by a lack of rhythmicity in the total sucking activity and or an inability to synchronize sucking, swallowing, and breathing.[1–3] This can place some preterm infants at an increased risk for dysphagia, characterized by aspiration or laryngeal penetration, resulting in complications such as pneumonia, respiratory disease, or poor growth [4–8]. Approximately 30% of very-low-birth-weight preterm infants will be diagnosed with dysphagia [9–13]. Given this prevalence, appropriate evaluation and treatment of dysphagia are of clinical importance to improve the medical outcomes of these at-risk preterm infants. The current conventional therapeutic options for preterm infants with dysphagia include the use of slow-flow nipples, positioning the infant in an elevated side-lying posture, pacing the infant’s sucking bursts, and thickening feeds. [14–24] However, these interventions may not always yield successful outcomes. Thickened feeds will alter the milk boluses’ shape, texture, and size, [22–25] which will stimulate the touch, pressure and stretch mechanoreceptors within the pharynx, increasing the sensory signals to the brain that will trigger more efficient swallowing movements. However, this method has its limitations, especially with breast milk, since enzymes in human milk can alter the desired consistency. [25,26]

A promising approach for adults with dysphagia involves using cold foods or liquids to stimulate a safer swallowing process. [27] Similar to the effect of thickened feeds, it is theorized that cold liquid increases sensory input to the brain by stimulating the sensory thermo-receptors in the pharyngeal area via cation channels expressed in the visceral afferents of the cranial nerves. This will increase the stimulation of the sensory receptors within the pharyngeal mucosa, triggering more efficient and safer swallowing movements. [27–29] Our research group was the first to demonstrate that short-duration feeding (5 swallows) with cold barium reduces aspiration occurrence from 71% to 26% in preterm infants diagnosed with dysphagia using videofluoroscopy. [30] Despite these encouraging results, the safety of using cold milk throughout the entire feeding session needs validation before it can be widely adopted. Moreover, reducing the risk of penetration and aspiration through the use of cold milk may not necessarily result in enhanced feeding performance. Studies in full-term and preterm infants have shown the safe practice of feeding cold milk. [31–34] These studies revealed tolerance to cold feeding with no significant adverse effects on the infants’ sleep patterns, vocalizations, motility, intake, feeding behavior, weight gain, temperature, frequency of regurgitation, or gastric emptying time. While previous studies demonstrated the safe use of cold feedings, [31–34] there are concerns that cold feeding will induce digestive malfunction [35,36] or cold stress associated with an axillary or rectal temperature of 36.0°C–36.4°C [37] and physiological manifestations. Furthermore, preterm infants are at an increased risk of necrotizing enterocolitis and hypothermia, which have been linked to increased risk of adverse neonatal outcomes. [38] Potential physiological reactions to cold stimuli include respiratory distress, desaturation, and altered digestive functioning, resulting in delayed gastric motility, increased gastric residuals, and emesis [36,39]. It is also unknown if feeding cold milk will negatively affect the preterm infants’ mesenteric blood flow as a sign of potential digestive malfunctioning or adversely affect their feeding performance. Our study aims to assess whether cold milk feedings could lead to cold stress, alter mesenteric blood flow, or adversely impact the feeding performance in premature infants with uncoordinated feeding patterns. We hypothesize that cold milk feedings will be well-tolerated without inducing cold stress or significant changes in mesenteric blood flow. Additionally, we expect that cold milk will not adversely affect the feeding performance of these infants.

## Materials/Subjects and Methods:

### Participants:

NYU Langone’s institutional review board (IRB) approved this study, and informed written consent was obtained before enrollment. The study was performed in accordance with the Declaration of Helsinki, was registered with clinicaltrials.gov and is not intended to report on long-term outcomes. All participants received care in the Neonatal Intensive Care Unit (NICU) at NYU Langone Hospital – Long Island. The study included preterm infants (< 37 weeks gestational age at birth) with a post-menstrual age (PMA) of 34–42 weeks at the time of enrollment and diagnosed with uncoordinated feeding pattern, as determined by the Quality Score from the Infant-Driven Feeding Scales (IDFS) [40] performed by the nursing/medical team. All infants involved were maintaining normothermia in an open crib. Infants with intra-uterine growth retardation or a history of gastrointestinal pathology, including necrotizing enterocolitis, gastroschisis, malabsorption, diarrhea, or omphalocele, were excluded from the study. Infants withdrawing from maternal drug use were also excluded from this study as these infants often demonstrate neurobehavioral abnormalities such as irritability, hyperactivity, tremors, high-pitched crying, and excessive sucking, which may alter their feeding patterns. [41] There were no restrictions with regard to gender, race or ethnic origin.

Participants’ demographic information collected included gender, race, gestational age at birth, birth weight, post-menstrual age (PMA), chronological age at the time of the study enrollment, and clinical information, such as feeding milestones and medical diagnoses.

### Methods:

All participants were assessed under two oral feeding conditions – room temperature milk and cold milk using an experimental, randomized crossover design. The order of feeds was randomized by flipping a coin. Condition A (control) indicates the infant’s typical milk feeding (formula or breastmilk) at room temperature (RT, defined as temperature between 68–77 °F). Condition B (intervention) indicates the same infant’s typical milk feeding (formula or breastmilk) fed at a cold temperature (CT, defined as the temperature between 39–48 °F). All participants were fed by an experienced research team member at the bedside. Both feeding conditions were performed within 12 hours of each other to reduce the effect of confounding factors. Participants were held in an elevated side-lying position [16,42] using a slow-flow nipple. Infants were fed following an infant-driven approach. When the infant demonstrated signs of stress or intolerance, their nutritive sucking pattern was interrupted by the feeder by either tilting the bottle down or removing the nipple from their mouths. This allowed the infant an opportunity to pause and breathe or to suck on an empty nipple.

Several procedural controls were used to ensure consistency across all feeding conditions and participants. To achieve a cold temperature, the participant’s milk was kept in a refrigerator to allow the liquid to cool to approximately 39–48 °F. To achieve room temperature, the participant’s feeding was kept at the bedside for no more than 2 hours prior to the feeding assessment. Before each feeding, the milk temperature was measured with a liquid thermometer (Red Lantern TP3001 Digital Thermometer. Tianjin, China) and sanitized after each participant per our institution’s sanitization policies and procedures. When measuring gastric content temperatures, the syringe was held by its base to prevent heat transfer from fingers, through the plastic, to the milk.

### Safety Assessment:

Two safety measures were implemented to identify potential signs of cold stress development. Before and after each feeding session, axillary temperatures were taken using a digital thermometer. The gastric content temperature was recorded for infants with a nasogastric tube (NGT) in place during oral feeding. However, for those without an NGT, this step was omitted to prevent the invasive procedure of inserting an NGT solely for the purposes of the study.

Before the start of each feeding session, the proper placement of the infant’s NGT in the stomach was verified by auscultation using a neonatal stethoscope. After confirming the NGT’s correct placement, the infant was fed, and the data regarding their feeding performance was collected. Immediately after each feeding condition, 2 ml of gastric content was extracted via the NGT using a 3 ml syringe. The gastric content’s temperature was determined using a digital thermometer. For Condition A, the gastric content’s temperature served as the baseline temperature. For Condition B, the temperature of the gastric content was checked at 1, 10, and 30 minutes post-feeding to track when it reverted to the original baseline temperature.

Doppler ultrasound (Sonosite Edge Ultrasound system, Sonosite, Inc, WA) was used to measure superior mesenteric artery (SMA) blood flow parameters as markers for intestinal blood flow. Peak systolic velocity (PSV) and end-diastolic velocity (EDV) were measured directly, and other indices of SMA mean velocity, such as Resistive index (RI) was calculated [RI = (PSV-EDV)/PSV] [43,44]. For each participant, a Doppler ultrasound was conducted six times. The intestinal blood flow measurements were initially taken one hour before each feeding session, then repeated at 30 and 60 minutes following the end of each feeding. These measurements were performed at the bedside with the participant in a supine position. To ensure consistency, all measurements were taken twice and performed by the same operator.

### Feeding Performance Assessment

We adopted objective and subjective methods of measuring feeding performance to evaluate the infants’ feeding skills during both feeding conditions. These measurements were captured and documented at 5-minute intervals throughout the feeding. The 5-minute intervals commenced at the moment the infant latched onto the nipple and began their first sucking burst. From the initiation of sucking, the timer was set to run continuously for 5 minutes, regardless of any pauses in sucking by the infant. The only instance where the timer was stopped before reaching the 5-minute mark was if the infant demonstrated clear signs of physiological instability. This 5-minute interval approach for data collection was chosen to enhance the accuracy of the subjective evaluations.

The objective measure of the Oral Feeding Skills score (OFS) [45] was administered during both feeding conditions. We collected the following data: a) The percentage of the volume consumed within the initial five minutes, b) The total volume that was prescribed, c) The overall volume ingested throughout the entire feeding session, and d) The total duration of active feeding. The OFS score was calculated in accordance with Lau and Smith [45] to achieve a score for Proficiency (% volume taken at 5 min/volume prescribed) and Rate of transfer (ml/min).

The subjective measure of suck-swallow-breathe coordination was scored within each 5-minute interval according to the Quality Score of the IDFS. [40] The IDFS used an ordinal measure of 1–5, with 1 being the best coordination and 5 being the worse coordination. [40] The IDFS is recommended as a valid and reliable tool to ease the safe and successful development of oral feeding skills in preterm infants and to plan evidence-based interventions. [46] The other two subjective measures, stress cues and the need for pacing, were rated within every 5-minute interval using an ordinal measure of 0–3 using a scoring system created for the purposes of this study, the Stress Scale and the Pacing Scale (Table 1). The Stress Scale identified stress cues in accordance with Als’ Synactive Theory, including cues specific to the motoric, behavioral and attention/interaction subsystems, [45] and the Pacing Scale is substantiated by previous research as well.[18–21] Additionally, the presence of any cardio-respiratory events, including apneas, bradycardias, or oxygen desaturations, [47] were detected using the Life Scope TR monitor. (Nihon Kohden America, Foothill Range, CA).

### Statistical Analyses:

Demographic characteristics were summarized using the median (Interquartile Range) or frequency (percentage) as appropriate. Continuous variables were assessed for normality using the Kolmogorov-Smirnov test, histogram, and Q-Q plot. Changes (RT vs. CT) for the paired data were computed for each clinical outcome and presented using mean difference along with the 95% confidence intervals. Data were compared between RT and CT using paired sample t-test for normally distributed data and Wilcoxon signed-rank test for variables that do not follow Gaussian distribution.

For ultrasound data, changes from the baseline were computed at 30 minutes and 60 minutes for both RT and CT, then the mean difference of the changes was computed along with 95% confidence intervals. The sample size was not statistically driven; however, the current sample size follows the recommendation to have at least 12 participants per group in pilot studies. [48] SAS 9.4 (SAS Institute Inc, Cary, NC) was used to analyze data, and statistical significance was assumed if p<0.05.

## Results:

A total of 26 infants were enrolled, including 5 infants who underwent pre- and post-feeding Doppler ultrasound assessment to measure mesenteric blood flow. As shown in Table 2, 26 study participants were born preterm (median gestational age at birth 31 weeks and 6 days). Participants’ median day of life and PMA at the time of the study were 28 days and 36 weeks and 1 day, respectively. All participants had experienced oral feeding before being enrolled in this study, with a median of 8 days of partial oral feeding at the time of the study. We selected infants <30 weeks gestation to perform the Doppler measurement of the mesenteric blood flow, since this gestational age is at higher risk of developing NEC. Table 3 demonstrates the demographics for the 5 infants who underwent Doppler for Mesenteric Blood Flow measures.

### Safety Assessment:

There was no reduction in participants’ axillary body temperature after CT feeding compared to RT feeding (p = 0.618), as demonstrated in Table 4. Gastric temperature measurements were only conducted in subjects who already had a nasogastric tube (NGT) in place at the time of the study (n=7). As shown in Table 4, by the time the cold milk reached the stomach (one minute after feeding), it had warmed up from its initial temperature of 68–77 °F to an average temperature of 83.3 °F. The temperature of the gastric content following CT feeding was only 3.4 degrees lower than that following RT feeding one minute after feeding, yet this difference reached statistical significance (Table 4). Additionally, no cold stress reactions were observed in any of the infants fed cold milk. This includes the absence of tachypnea, respiratory distress, desaturation, increased episodes of apnea and bradycardia, or changes in digestive function, such as increased gastric residuals and emesis.

There were no significant differences in the mesenteric blood flow Doppler findings between CT and RT feeding (Table 5). Both groups (RT and CT) showed elevated PSV and EDV measurements at 30 minutes, with a mean increase of 13.5 and 10.7cm/second from the pre-feeding baseline. At 60 minutes, both PSV and EDV were lower than 30-minute measurements but remained higher than pre-feeding measurements (mean increase of 5.2 and 5.1cm/sec from baseline). Resistive indices were unchanged at 30 and 60 minutes compared to pre-feeding baseline measurements. Since the initial 5 cases did not reveal any impact of cold milk on mesenteric blood flow, additional Doppler studies were not conducted to avoid unnecessary procedures for the enrolled patients.

### Feeding Performance Assessment

A total of 21 infants completed both pre and post-feeding performance assessments. There were no statistically significant differences in OFS Levels for infants fed CT milk compared to those fed RT milk (p=0.734), as indicated in Table 6. Additionally, other objective measures of proficiency (% volume taken at 5 min/volume prescribed), Rate of transfer (ml/min), and overall transfer, showed no significant differences between the two feeding conditions. Similarly, subjective measures related to stress cues and pacing (Table 6) did not demonstrate any significant differences between CT and RT feeding conditions. However, the coordination of sucking, swallowing, and breathing, as evaluated by the Infant Dysphagia Feeding Scale (IDFS), showed statistical improvement in the group fed CT compared to RT milk (p = 0.001). During the RT feeding condition, the average IDFS score for Quality was 3.2 (SD 0.7), while for the CT condition, it was 2.6 (SD 0.9), as shown in Table 6.

## Discussion:

This pilot study demonstrates that feeding preterm infants with cold milk can be safely tolerated without any clinical adverse events. There were no clinically significant alterations in axillary temperature or mesenteric blood flow parameters following CT milk feedings compared to RT milk feedings. Cold milk feedings did not adversely impact feeding performance. On the contrary, cold milk may improve some aspects of feeding performance in preterm infants who exhibit uncoordinated feeding patterns. This improvement was observed using the IDFS scoring, which indicated better coordination of sucking, swallowing, and breathing. To the best of our knowledge, this is the first study to investigate the safety of cold milk feeding in preterm infants.

Although the average temperature of gastric content during CT milk feeding in our study was statistically lower than during RT milk feeding, this difference does not necessarily imply clinical significance. This is evidenced by the absence of changes in mesenteric blood flow or any signs of feeding intolerance induced by the CT milk. The study by Bissinger et al. explored thermoregulation in very low birth weight (VLBW) infants, highlighting that the optimal ambient temperature to support their thermal stability ranges between 71°F and 80°F. [36] Although their research focused on external cold exposure while our study investigated the effects of internal cold exposure from cold milk feeding, our findings align with theirs. Our results demonstrated that the exposure to cold milk did not induce a critical decrease in stomach temperature (with an average of 82.9 °F, which is higher than the optimal ambient range) and consequently did not trigger a cold stress response. This suggests that internal exposure to cold from milk feeding, similar to external exposure, does not adversely affect the thermal stability of VLBW infants. [36]

For preterm infants the standard practice is to administer gavage feedings of either human milk or formula at room temperature, typically within the 68–77 °F range, until they are capable of oral intake. This approach indicates that introducing milk at ambient room temperature into the stomach is generally well-tolerated without gastrointestinal adverse effects. Our study showed that when cold milk is fed orally to preterm infants, it reaches the stomach at an approximate temperature of 83.3 °F, which is above the typical room temperature range (68–77 °F) that is used when milk is introduced directly into the stomach via NGT or OGT feeding methods. [37] Since it was not the focus of our study, we did not investigate the effects of introducing cold milk directly to the stomach via NGT. Our findings are consistent with a study conducted on adult volunteers, which observed that gastric temperatures returned to within 1 degree of core body temperature within 30 minutes of consuming a cold drink. [46] In our study population exposed to cold milk, there were no gastrointestinal adverse events, such as vomiting, feeding intolerance, increased gastric residuals, or alterations in stool consistency or frequency. These results align with the guidelines from the Centers for Disease Control and Prevention (CDC), which state that babies can safely consume cold milk. [49]

One possible concern with cold milk feeding is its potential to affect intestinal blood flow, theoretically causing mesenteric vasoconstriction. To address this concern, our research findings indicate that the impact of cold feeding on mesenteric blood flow in preterm infants is minimal. Specifically, no significant changes were observed in the Superior Mesenteric Artery (SMA) blood flow, as evidenced by Resistive Index (RI) measurements taken before, and then 30 and 60 minutes after CT milk feeding, compared to RT feeding.

Our previous research has demonstrated the efficacy of using cold milk for a short duration (limited to 5 swallows) in reducing penetration and aspiration in premature infants with dysphagia [30]. While using cold milk may lower the risk of penetration and aspiration, this does not automatically imply an improvement in overall feeding efficiency or the effectiveness of transferring milk. Although the primary aim of this current study was to investigate the safety of administering a full feeding of CT milk to preterm infants, it also examined if CT milk would alter the infants’ feeding performance. Our findings indicate that cold milk feedings did not negatively impact feeding performance. Moreover, some preterm infants even showed an improvement in feeding performance when given CT as opposed to RT milk. The observed potential enhancement in the coordination of sucking, swallowing, and breathing with CT milk is supported by research in adults indicating that cold liquids enhance sensory stimulation to the thermal receptors in the oral cavity and hypopharynx, which in turn may increase coordinated motor activity. [24,25,46–48] However, these findings of improved feeding performance with CT have to be interpreted in the context of the subject characteristics at the time of the study. At the time of the study, the median PMA was 36wk 1d, with a median of 8 days of oral feeding experience and consuming 50% of the feeding volume orally. This suggests that recommending cold milk solely to influence the infants’ feeding performance needs further research to confirm efficacy. However, CT milk should not negatively impact the infant’s feeding performance for those with possible aspiration/penetration.

We also observed a lack of agreement between the objective and subjective feeding assessment tools. The OFS follows a volume-driven approach, emphasizing feeding intake quantities and suggests that feeding performance depends on the amount and speed of liquid ingested from a bottle. [50] In contrast, the IDFS aligns with an infantdriven approach, placing more importance on the quality of the feedings and aiming for infants to engage in feeding without distress or disorganization. [18,20,40,51] Although we assumed both measurements would yield similar results, cold milk feeding minimally affected OFS but significantly improved IDFS. We found that some infants in our study scored well on the OFS ingesting larger volumes quickly but did not display a coordinated sucking pattern, as indicated by a poorer score on the IDFS. This inverse relationship is consistent with other research on the subject [15,17,52–54] and supports a co-regulated, cue-based, and infant-driven approach. Recent studies have shown that using an infant-driven approach accelerates feeding advancement and shortens hospital stays for premature infants, especially in those born at ≥28 weeks. [55,56] Additionally, this approach reduces hospital readmissions within the first year of life and improves long-term feeding outcomes while decreasing the frequency of feeding therapy consults in the NICU. [57,58] These positive outcomes are attributed to feedings focused on enjoyable experiences characterized by physiologic stability, motor organization, and coordinated suck, swallow, and breathe patterns. [51,54,59–61]

A limitation of this study is the small sample size; however significant correlations were noted despite including only 26 participants. Another challenge was the absence of video recordings for each feeding session. The ability to review these feedings retrospectively could have allowed for blind inter-rater evaluations, thereby strengthening the validity and reliability of our findings. While the main objective of our research focused on investigating the safety of cold milk feeding, further research should build upon these findings to enhance our comprehension of cold milk’s efficacy as a clinical tool for dysphagia. Additionally, future studies should explore the effects of cold milk in conjunction with other treatment techniques, such as thickened milk, to enhance the available therapeutic options.

## Conclusion:

Our research findings conclude that cold milk feedings in preterm infants were safely tolerated without clinically significant changes in axillary or gastric temperature or mesenteric blood flow compared to room temperature feedings. Additionally, cold milk feedings did not adversely affect feeding performance in preterm infants with uncoordinated feeding patterns. These results highlight the potential for further comprehensive studies to explore the use of cold milk as an effective therapeutic approach for addressing feeding and swallowing difficulties in preterm infants. Given the safety profile of cold milk feeding demonstrated in this study, we suggest that cold milk feeding could be considered a viable option for preterm infants with dysphagia after all other alternatives have been evaluated. However, careful assessment, individualization, and close monitoring are required to ensure its effectiveness for each infant.

## Figures and Tables

**Table 1.** Scoring Rubric for Subjective Scales

	0	1	2	3
**Stress Scale**	No behavioral stress cues observed	1–3 behavioral stress cues observed	4–10 behavioral stress cues observed	More than 10 behavioral stress cues observed
**Pacing Scale**	No pacing is offered. Infant self-regulated suck-swallow breathe coordination	Pacing is offered 1–3 times	Pacing is offered 4–10 times	Pacing is offered >10 times

**Table 2:** Demographics and clinical characteristics of infants receiving pre and post feeding safety measures and performance measures (n=26)

Variable	Frequency (Percentage) or Median (IQR)
Female sex, n (%)	15 (57.7%)
Gestational age at birth, median, weeks/days	31 6/7 (28 2/7, 32 4/7)
Birth weight, median, grams	1625 (837, 1966)
Day of life at the start of the study, median	28 (16, 73)
Post Menstrual Age (feeding introduction), median, weeks/days	34 5/7 (34, 36 6/7)
Post Menstrual Age at the start of the study, median, weeks/days	36 1/7 (35 1/7, 38 5/7)
Days of oral feeds at the start of the study, median	8 (5, 13)
Percent oral feed at the start of the study, median	50 (40, 70)

**Table 3.** Demographics and clinical characteristics of infants undergoing Ultrasound assessments (n=5)

Variable	Frequency (Percentage) or Median (IQR)
Female sex, n (%)	3 (60%)
Gestational age at birth, median, weeks/days	26 5/7 (25, 29 2/7)
Birth weight, median, grams	615 (600, 635)
Day of life at the start of the study, median	27.5 (16, 73)
Post Menstrual age (feeding introduction), median, weeks/days	37 2/7 (36 4/7, 37 5/7)
Post Menstrual Age at the start of the study, median, weeks/days	39 2/7 (38 5/7, 39 5/7)
Days of oral feeds at the start of the study, median	10 (2, 14)
Percent oral feed at the start of the study, median	27 (7, 30)

**Table 4.** Safety outcomes comparisons between RT and CT (n=21)

Variable	Mean(SD) RT	Mean(SD) CT	Change(RT-CT) 95% CI	P-value
Body Temp (°F) PRE feeding	98.3(0.3)	98.3(0.2)	−0.1 (−0.2, 0.1)	0.379
Body Temp (°F) POST feeding	98.2(0.3)	98.3(0.3)	0 (−0.2, 0.1)	0.618
Gastric Temp (°F) 1-min POST feeding §	86.7 (0.4)	83.3 (1.1)	3.6 (2.0, 5.2)	**<0.001**
Gastric Temp (°F) 10-min POST feeding §	86.7 (0.4)	82.9 (1.1)	4.0 (2.3, 5.4)	**<0.001**
Gastric Temp (°F) 30-min POST feeding §	86.7 (0.4)	84.2(1.0)	2.7 (1.3, 4.1)	**0.003**

P values are from Paired t-test for normally distributed variables and Wilcoxon signed-rank test for non-normally distributed variables. SD-Standard deviation, F- Fahrenheit, § effective sample size, n=10, Bold: indicates significant P value.

**Table 5.** Mesenteric Blood Flow Doppler Findings (n=5)

Mean based			
Variable	Change from baseline RT Mean(SD)	Change from baseline CT Mean(SD)	(CT-RT) Difference Mean (95% CI)*
Changes at 30 minute			
PSV	13.5(8.4)	10.7(3.8)	−0.1(−11, 10.7)
EDV	3.5(3.4)	0.8(2.8)	−1.9(−7.2, 3.3)
RI	0(0)	0(0)	0(0, 0.1)
Changes at 60 minute			
PSV	5.2(5)	5.1(9.7)	1.1(−10, 12.3)
EDV	0.4(1.1)	−0.1(0.6)	−0.5(−3.1, 2.1)
RI	0(0)	0(0)	0(0, 0.1)

* n=4

Table shows ultrasound measures obtained for superior mesenteric artery blood flow parameters – Peak systolic velocity (PSV) and End diastolic velocity (EDV) were measured, and Resistive indices (RI) were calculated. Measurements were obtained before feeding, after 30 minutes and after 60 minutes of feeding for both room temperature (RT) and cold temperature (CT) feedings for each subject. Peak systolic velocity (PSV), End-diastolic velocity (EDV), Resistive Index (RI), Room Temperature feeding (RT), Cold Temperature feeding (CT), Standard deviation (SD), 95% Confidence Interval (95% CI)

**Table 6.** Feeding performance outcomes comparisons between RT and CT (n=21)

Variable	Mean(SD) RT	Mean(SD) CT	Change(RT-CT) 95% CI	P-value
Total Length of Feeding (min)	12.4(4.6)	14.3(4.6)	−1.9(−4.8, 1.1)	0.203
Total volume ingested for feeding (ml)	25.6(16.7)	28(14.4)	−2.4(−6.6, 1.7)	0.238
OFS Level Ɨ	2.3(1.3)	2.2(1.2)	0.1(−0.4, 0.6)	0.734
Proficiency	23.5(12.9)	25.5(15.3)	−2(−7, 3)	0.425
Rate of Transfer	2(1.2)	2(1)	0(−0.3, 0.4)	0.781
Overall Transfer Ɨ	54(35.8)	58.7(31.3)	−4.7(−13.5, 4.1)	0.147
IDF-Quality Raw Score Ɨ	3.2(0.7)	2.6(0.9)	0.6(0.2, 1)	**0.001**
Pacing Scale Ɨ	1.4(1)	1(0.9)	0.4(0, 0.8)	0.057
As. Bs. Ds. Ɨ	0.3(0.9)	0.2(0.7)	0.1(−0.1, 0.4)	0.375
Stress Scale Ɨ	1.6(0.6)	1.7(0.8)	0(−0.3, 0.3)	0.933

P values are from Paired t-test for normally distributed variables and Wilcoxon signed-rank test for non-normally distributed variables; SD-standard deviation; Ɨ not normally distributed; Bold: indicates significant P value; RT, room temperature; CT, cold temperature; OFS, Oral Feeding Skills; AsBsDs, apneas, bradycardias, desaturations.

## Data Availability

The datasets generated from the data analysis are not available publicly due to patient privacy restrictions and regulations. A limited, de-identified dataset may be available from the corresponding author upon reasonable request.
